# Cytochrome P450 oxidoreductase deficiency caused by R457H mutation in *POR* gene in Chinese: case report and literature review

**DOI:** 10.1186/s13048-017-0312-9

**Published:** 2017-03-14

**Authors:** Yang Bai, Jinhui Li, Xiaoli Wang

**Affiliations:** Department of Endocrinology and Metabolism, Institute of Endocrinology, Liaoning Provincial Key Laboratory of Endocrine Diseases, The First Affiliated Hospital of China Medical University, Nanjing North Street, NO 155, Shenyang, 110001 People’s Republic of China

**Keywords:** Case report, Cytochrome P450 oxidoreductase, Mutation, Congenital adrenal hyperplasia, POR deficiency

## Abstract

**Background:**

Cytochrome P450 oxidoreductase deficiency (PORD) is a rare disease exhibiting a variety of clinical manifestations. It can be difficult to differentiate with other diseases such as 21-hydroxylase deficiency (21-OHD), polycystic ovary syndrome (PCOS) and Antley–Bixler syndrome (ABS). Nearly 100 cases of PORD have been reported worldwide. However, the genetic characters and clinical management are still unclear, especially in China.

**Case presentation:**

In this study, we report a 27-year-old female Chinese patient who first presented with amenorrhea and recurrence of large ovary cyst. She was misdiagnosed with PCOS and non-classical 21-OHD due to ovary cysts and elevated 17-hydroxy-progesterone. The patient’s complaining of a mild difficulty of bending the metacarpophalangeal joints reminded us to consider PORD, which usually presents with skeletal deformities and sexual dysfunction. The diagnosis of PORD was confirmed by genetic analyses, which showed the patient harboring a homozygous missense mutation in the *POR* gene (R457H) and her parents carrying the heterozygous mutation. The patient was treated with low-dose corticosteroids and estrogen/progesterone sequential therapy, and her ovarian cyst gradually reduced with regular menstruation in the follow-up. Moreover, the clinical and genetic characteristics of 104 previously reported PORD cases were also summarized and analyzed.

**Conclusions:**

PORD is a very rare disease which can be easily misdiagnosed in mild cases. Clinicians should keep in mind of this disease in patients with sexual dysfunction, especially combined with special skeletal deformities. Our data could provide a consciously understanding of this disease for clinic practicers. Low-dose corticosteroids combined with estrogen/progesterone sequential therapy will be effective in PORD patients with recurrence of large ovary cyst. The fact that the reported PORD patients in China carrying an identical variant R457H in *POR* gene also give us a viewpoint that R457H mutation in POR gene maybe important in causing PORD in Chinese as same as in Japanese.

## Background

**Fig. 1:**
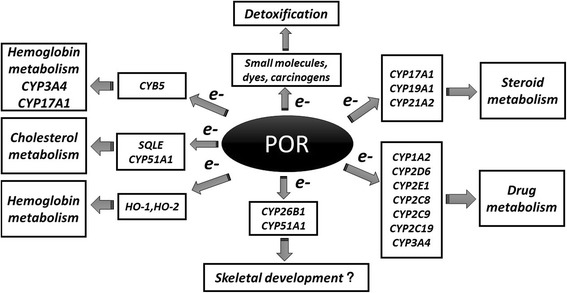
POR involvement in various biological pathways. POR donates electrons to all P450 enzymes, and at the same time, provides electrons for some proteins and small molecules, which involved in numerous biological activities such as biosynthesis, cholesterol metabolism, sex hormone metabolism and the metabolism of drugs and toxins

Cytochrome P450 oxidoreductase (POR) is encoded by the *POR* gene. POR transfers electrons from NADPH to cytochrome P450 enzymes for drug and toxin metabolism as well as steroid hormone synthesis (Fig. 1) [1]. Since P450 enzymes are involved in the catalysis of many substrates, POR deficiency (PORD) (OMIM: 613571 and OMIM: 201750) can lead to disorders of steroid hormone, drug and toxin metabolism, to various extents. PORD resulting from mutations in the *POR* gene has been reported by different teams since 2004 [2–6]. Currently, nearly 100 cases have been reported worldwide (Table 1). However, the treatment method of this rare disease is not clear due to the limited number of patients.

**Table 1:** Summary of the PORD cases reported in the literature

Number of cases	Ethnicity (Japanese: European:others)	Gender (female:male)	Age (infant:adult)	POR mutation	skeletal deformities	Gonadal deformities	Hormonal abnormalities or delayed puberty	Maternal virilization	Adrenal insufficiency or crisis	Ovarian cysts	References^a^
1	0:0:1	1:0	1:0	2/2	0	1	1	0	–	–	[25]
1	0:0:1	1:0	1:0	2/2	1	1	1	1	1	1	[26]
2/3	0:2:0	1:0	2:0	4/4	2	2	–	0	–	–	[27]
1	0:1:0	0:1	0:1	2/2	0	1	1	–	0/1	–	[28]
30^b^	0:26:4	18:12	23:7	54/60	27	22	28	1/3	24/27	4/4	[16]
2	0:0:2	2:0	2:0	4/4	2	2	2	–	2	0	[29]
4	0:0:4	3:1	2:2	8/8	3	3	4	1/1	4	0	[30]
35^c^	35:0:0	19:16	21:14	70/70	28	26	35	17/35	11/21	8/18	[13]
1	0:0:1	1:0	1:0	2/2	1	1	1	0	1	–	[31]
4	0:0:4	0:4	4:0	8/8	0	4	4	0	4	–	[32]
1	0:1:0	1:0	1:0	1/2	1	1	1	0	–	–	[33]
19/38^d^	3:11:5	6:10	19:0	34/38	19	12	10	–	2/5	–	[11]
3/4^e^	0:3	1:2	2:1	5/6	2	2	3	–	1/1	1/1	[6]
104	38:46:20	54:46	79:25	94.2%	82.7%	75%	89.2%	40.8%	74.6%	46.7%	

^a^Because some of the cases in the older literature have also been described in newer reports, the order of the literature descends from newer to older, and repeated cases have been omitted

^b^There were 30 cases reported in this study with 17 cases repeated from older reports

^c^This study reported 35 cases, and among them, 23 cases were repeated from older reports

^d^The literature reported 38 cases. In the 32 cases who were suspected to have ABS, *POR* gene mutations were identified in 19 cases; for *FGFR2*, three mutations were identified in nine cases; no gene mutations were found in four cases; and another six cases were Beare–Stevenson syndrome patients

^e^This study reported PORD for the first time, in which one case was repeated in a subsequent large-sample report, so it was omitted

PORD can cause reduced activities of multiple enzymes participating in the synthesis of both adrenal and gonadal steroid hormones, which further leads to the dysfunction of the sex hormones and a decrease in glucocorticoids. Therefore, the disease is a type of special congenital adrenal hyperplasia (CAH). PORD is very rare and exhibits a variety of clinical manifestations, such as oligomenorrhea and ovarian cysts which resemble polycystic ovary syndrome (PCOS), elevated 17-hydroxy-progesterone (17OHP) which resembles 21-hydroxylase deficiency (21-OHD), and skeletal deformities which resemble Antley–Bixler syndrome (ABS). Therefore, in some cases, it can be difficult to differentiate PORD from PCOS, 21-OHD and ABS. However, PORD usually doesn’t exhibit hyperandrogenism which differs from PCOS and 21-OHD. In this study, we report a female adult Chinese PORD case who was misdiagnosed many times, and analyze the clinical presentation and genetic character of the patient and her parents.

## Case presentation

### Patient characteristics

**Fig. 2:**
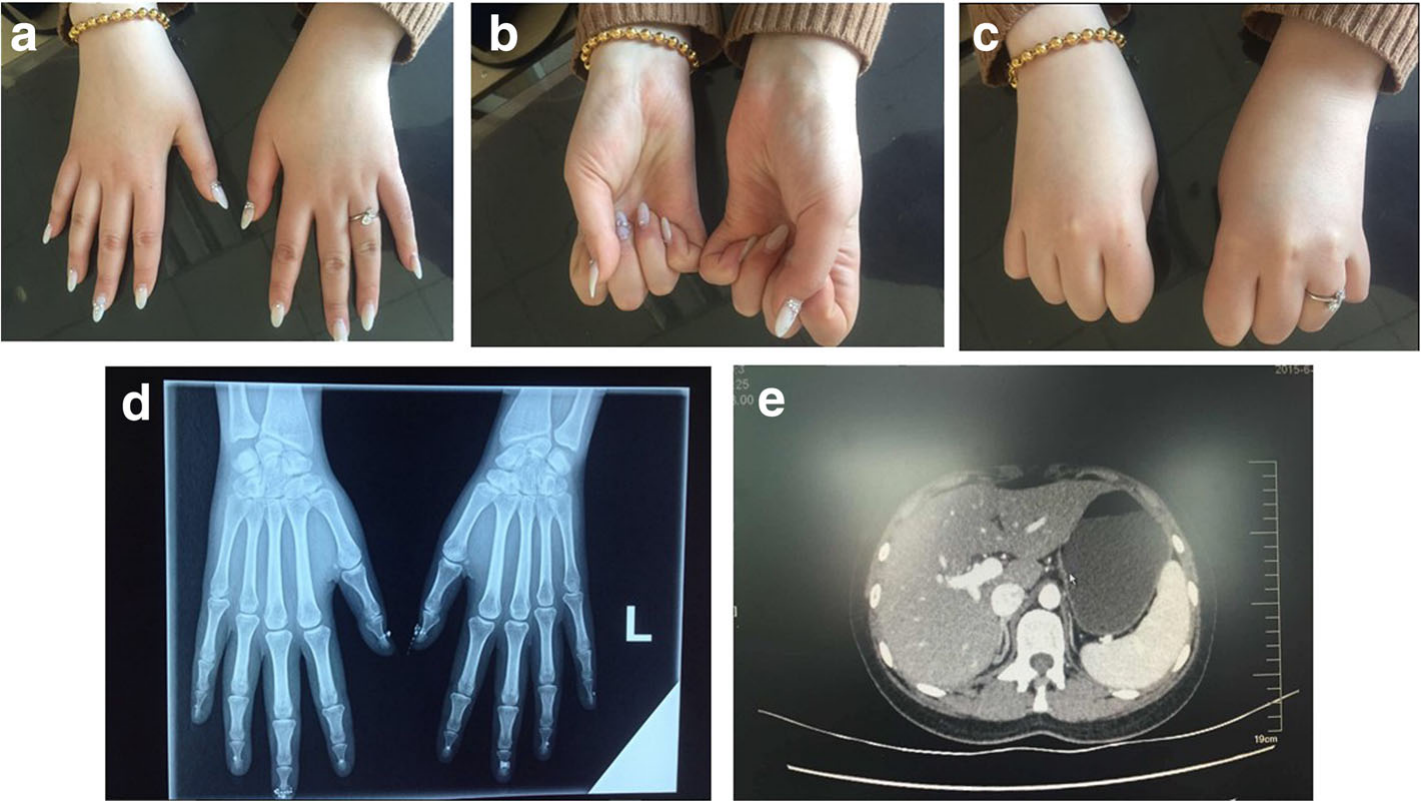
The appearance of the hands of the patient, hand DR and adrenal CT scans. **a**-**c** shows patient’s difficulty of bending the metacarpophalangeal joints, however her hand DR (**d**) suggested there were no bone abnormalities. The adrenal CT scan (**e**) showed there were no obvious hyperplasia changes

A 27-year-old female (46,XX) patient with normal cognition visited the Endocrinology Clinic of 1^st^ affiliated hospital of China Medical University (Shenyang) for amenorrhea with an unknown etiology. The patient had labial fusion when she was born and underwent plastic surgery afterward. During puberty, the patient underwent another surgery for an ovarian cyst. The patient was treated in another hospital as PCOS for several times resulting with mild remission of amenorrhea and recurrence of ovarian cysts. The patient was suspected of having 21-OHD due to increased 17OHP levels and came to our hospital for a genetic test for *CYP21A2* mutations. The patient did not have fatigue, loss of appetite or other symptoms and was the only child in the family. Her parents appeared to be normal. She was born by vaginal delivery, and the mother had no complaints of abnormal manifestations during the perinatal period or history of reproductive system diseases. Her parents are nonconsanguineous. Physical examination of the patient revealed the following characteristics: a height of 165 cm and weight of 55 kg; no hyperandrogenism symptoms like hirsutism or acne; and no purple stripes. The patient complained of difficulty of bending the metacarpophalangeal joints from childhood (Fig. 2a-c). There were no obvious abnormalities in the genitals.

### Clinical examination and testing

The ultrasound showed the presence of a 1.2 × 1.4 cm fluid area in the left ovary, and there was a 9.5 × 6.3 × 4.3 cm cyst in the right ovary behind the uterus. Digital radiography (DR) suggested there were no bone abnormalities (Fig. 2d). The adrenal CT scan showed there were no obvious hyperplasia changes (Fig. 2e). The laboratory test results of the patient and her parents are shown in Table 1. The levels of progesterone and 17-hydroxy progesterone of the patient were significantly increased. Androgen levels were not increased, and the basal level of cortisol in the morning was within the lower limit of the normal level. Cortisol was stimulated by the ACTH stimulation test. There were no abnormalities in the other measurements or in those of the parents.

### Gene testing

**Fig. 3:**
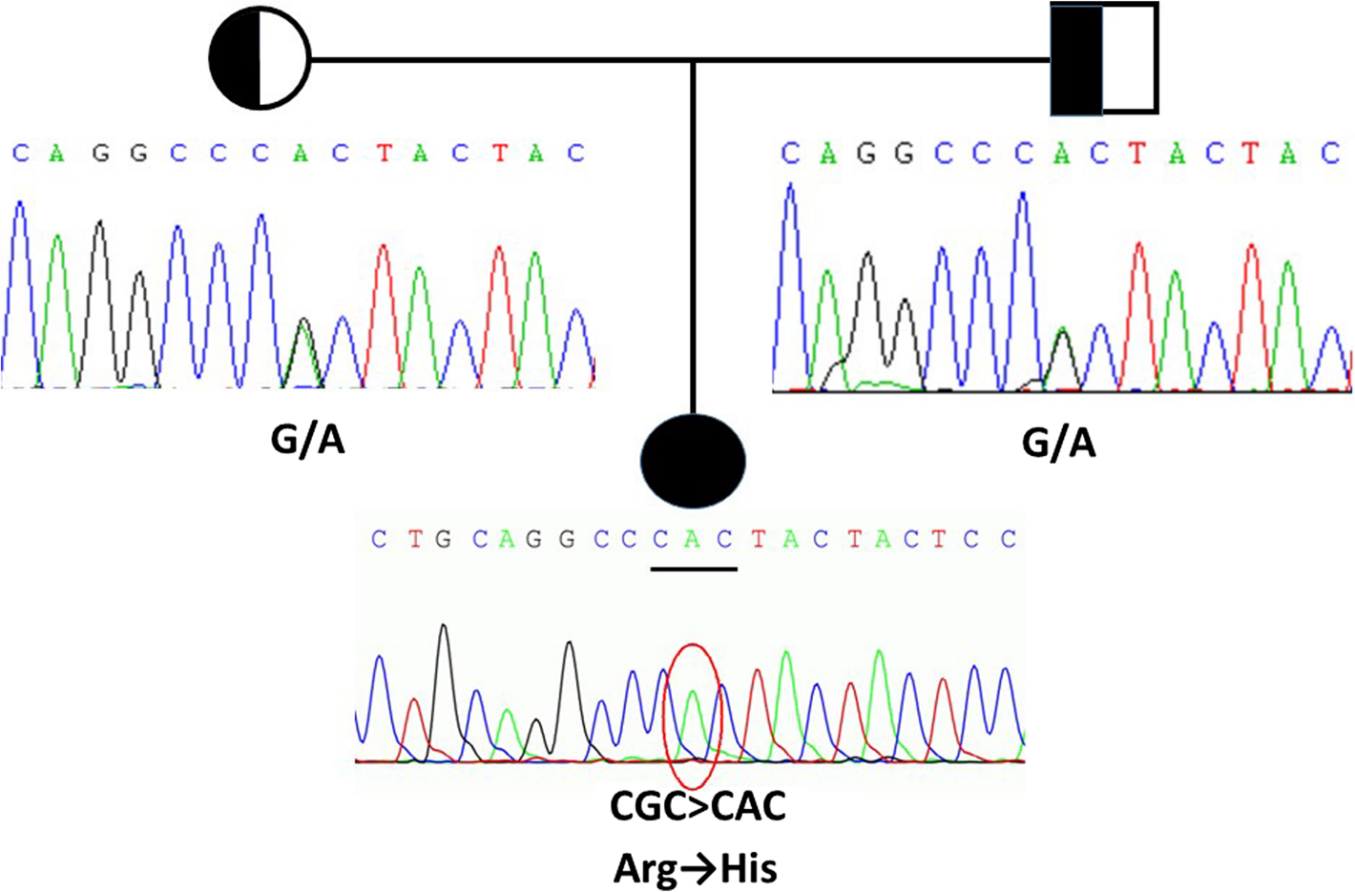
*POR* gene testing results of the patient and her parents. Exon 11 of *POR* harbored a homozygous mutation (c.1370G > A) which leads to a conversion of arginine at amino acid position 457 to histidine (R457H). The patient’s parents were both heterozygous carriers of this variant

*CYP21A2* (NM_000500) was tested first due to the suspicion of 21-OHD, but no mutations were found in the patient. Then the patient’s complaint about a mild difficulty of bending the metacarpophalangeal joints reminded us to consider PORD, which usually presents with skeletal deformities and sexual dysfunction, so *POR* (NM_000941) gene was tested secondly. Exon 11 of *POR*harbored a homozygous mutation (c.1370G > A) which leads to a conversion of arginine at amino acid position 457 to histidine (R457H). The patient’s parents were both heterozygous carriers of this variant (Fig. 3). Since the disease-causing homozygous mutations in POR gene were found, no further genetic analysis was performed.

### Treatment and follow up

Our patient once underwent surgery to remove a large ovarian cyst; however, since the diagnosis was unclear and hormone replacement therapy was not given, the ovarian cyst soon recurred. The patient was given low-dose corticosteroids twice a day (hydrocortisone 5 mg in the morning and 2.5 mg in the afternoon) and estrogen/progesterone sequential therapy in our hospital, and her ovarian cysts gradually decreased in size (the biggest ovarian cyst shrank to 3.5 × 3.3 × 2.3 cm) with regular menstruation in the following visits. The serum levels of ACTH, LH, 17OHP and P were declined accordingly (Table 2). No adverse effect of corticosteroids was found in the follow-up.

**Table 2:** Test results of the patient and her parents

	Father	Mother	Patient (46, XX)		
			Baseline	3 months after treatment	6 months after treatment
age (y)	55	54	27		
FT (pmol/L)	116.00 (55.05–183.50)	6.42 (0.77–33.03)	6.10 (0.77–33.03)	–	5.38
SHBG (nmol/L)	9.00 (13.00–71.00)	48.50 (18.00–114.00)	80 (18.00–114.00)	–	78
T (nmol/L)	27.70 (9.08–55.23)	<0.69 (0.69–2.15)	<0.69 (0.69–2.77)	–	<0.69
AND (nmol/L)	4.76 (1.05–11.52)	1.53 (2.09–10.82)	<1.05 (2.09–10.82)	–	<1.05
DHEA (umol/L)	1.70 (2.17–15.20)	1.95 (0.95–11.67)	0.76 (0.95–11.67)	–	0.82
E2 (pmol/L)	117.0 (73.4–206.0)	106.0 (73.4–110.0)	244.0 (73.4–587.0)	225	231
P (nmol/L)	1.17 (0.86–2.90)	<0.64 (0.64–3.20)	8.68 (0.64–3.60)	4.61	3.83
17OHP (ng/mL)					
Baseline	0.80 (0.50–2.10)	1.20 (0.13–0.51)	9.60 (0.10–0.80)	3.8	3.1
60 min after 25U ACTH iv	–	–	>20	–	–
LH (mIU/mL)	7.76 (0.80–7.60)	50.60 (11.3–398.00)	4.78 (1.10–11.60)	2.72	2.73
FSH (mIU/mL)	12.30 (0.70–11.10)	92.90 (21.70–153.00)	7.14 (2.80–11.30)	6.40	6.18
PRL (mIU/mL)	119.0 (40.0–530.0)	111.0 (40.0–530.0)	443.0 (40.0–530.0)	–	383.2
ACTH (pg/ml)	48.71 (7.20–63.30)	10.77 (7.20–63.30)	48.75 (7.20–63.30)	33.48	14.52
COR (nmol/L)					
Baseline	497.8 (171.0–536.0)	244.1 (171.0–536.0)	198.5 (171.0–536.0)	466.5	303.3
60 min after 25U ACTH iv	–	–	642.3	–	–

*T* testosterone, *FT* free testosterone, *AND* androstendione, *SHBG* sex-hormone binding globulin, *DHEA* dehydroepiandrosterone, *E2* estradiol, *ACTH* adrenocorticotropic hormone, *COR* cortisol, *LH* luteinizing hormone, *FSH* follicle-stimulating hormone, *PRL* prolactin, *17OHP* 17-hydroxyprogesterone, *P* progesterone

## Discussion

PORD is a rare variant of CAH disease. As early as 1985, a CAH case which had characteristics of both 21-OHD and 17α-hydroxylase deficiency (17-OHD) was reported; however, no mutations were found in the patient in *CYP21A2* or *CYP17A1*, two genes known to underlie these diseases [7]. In 2004, Fluck et al. reported that this new CAH disease was caused by a mutation in the *POR* gene, and they also showed that the mutation of *POR* could lead to a decrease in the activity of P450 enzymes including CYP21A2, CYP17A1 and CYP19A1 [6]. POR donates electrons to all P450 enzymes, and at the same time, provides electrons for some proteins and small molecules (Fig. 1). POR plays an important role in biological activities such as biosynthesis, cholesterol metabolism, sex hormone metabolism and the metabolism of drugs and toxins. In addition, the loss of POR enzyme activity varies with *POR* mutation type; thereby, PORD has a variety of clinical manifestations from menstrual disorders to severe hermaphroditism and skeletal malformations, and even fetal death. At present, around 100 PORD cases have been reported worldwide (Table 1). The proportion of female and male cases is roughly equal (54:46). Most of the reported patients are of Japanese or European descent (38:46). The majority are newborn or fetal cases (76%), and adults account for 24% reported cases. The primary clinical manifestations include abnormal hormone levels at birth or developmental delay after puberty (89.2%), hermaphroditism (75%), maternal virilization (40.8%), ABS-like skeletal abnormalities (82.7%), underlying adrenal insufficiency (74.6%) and ovary cysts in females (46.7%). Clinicians should always keep in mind of this disease in patients with sexual dysfunction, especially combined with special skeletal deformities.

Most of PORD patients presented with ambiguous genitalia (75%). The pathogenic mechanisms of this manifestation are associated with impaired syntheses of testosterone, dihydrotestosterone and conversions of the androgens to estrogens. These syntheses and conversions depend on CYP17A1, CYP19A1, CYP21A2, which require POR [8, 9]. In addition, maternal virilization (40.8%) is described in mothers during pregnancies of PORD fetus, and it is also due to placental aromatase (CYP19A1) deficiency [10].

Similar to 21-OHD, despite that the basal levels of ACTH and cortisol are generally normal in PORD patients, the ACTH stimulation test showed that most of the patients (74.6%) have insufficient adrenocortical reserve. Activities of different POR mutants for target enzymes would have a certain role in variability of adrenocortical insufficiency [11–13]. Patients with insufficient adrenocortical reserve may need long-term glucocorticoid replacement therapy [14], especially during disease, inflammation, stress or surgery. Mineralocorticoid dysfunction is not apparent in PORD patients. However, PORD patients who are homozygous for an A287P mutation have been found to have increased blood pressure (caused by an increase in deoxycorticosterone), which is similar to that seen in 17-OHD [15]. Thus, regular blood pressure monitoring is required for PORD patients.

Ovarian cysts were found in this patient and in other adult female patients (46.7%). If patients have a history of amenorrhea or anovulation, the disease is easily misdiagnosed as PCOS at primary medical units. The decrease in estrogen causes hypergonadotropic hypogonadism, which stimulates the growth of the ovaries. On the other hand, mutations in *POR* lead to the reduced activity of CYP51A1, thus reducing the synthesis of meiosis-activating sterol and further inducing a dysfunction in meiosis and the maturation of the oocytes. Under the above-mentioned “double hit”, compared with ovarian cysts associated with other hormone synthesis disorders, those in PORD are more difficult to control. Surgical treatment combined with corticosteroid and sex hormone replacement therapy is often required to prevent recurrence [14]. Our patient responded well to this therapy during follow-up.

Another important clinical feature of PORD is that there are different degrees of Antley-Bixler syndrome-like skeletal deformities (82.7%). Skeletal deformities include midface hypoplasia, craniosynostosis, foot and hand deformity, bony union of the large joints and femur bending. Some researchers have attempted to quantify these skeletal deformities and assess the severity according to quantitative indices. They found that compound heterozygous and homozygous mutations in the *POR* gene are associated with more serious skeletal deformities. This may also result from the effect of various mutations on the activity of related enzymes [16]. Our patient harbored a homozygous mutation but only had mild metacarpophalangeal joint contracture, which is not a typical skeletal deformity, showing the heterogeneity of this symptom in PORD. The pathogenic mechanisms of PORD-associated skeletal deformities may be associated with lanosterol 14α demethylase (CYP51A1) which involved in cholesterol biosynthesis [17] and retinoic acid which metabolized by microsomal CYP26 proteins [18].

P450 liver enzymes and heme oxygenase involving in drug metabolism need POR to provide electrons, therefore, the mutation of POR gene or associated SNPs may affect drug metabolism in human body [19, 20] and affect the progression of some diseases such as malaria and sepsis [21]. For example, the R457H mutation in this patient can inactivate the P450 enzymes including CYP1A2, CYP2C19, CYP2D6 and CYP3A4 which are important for drug metabolism [12, 22, 23]. Thus, there may be possible risks if the PORD patients are administered medications metabolized by these enzymes.

It has been reported that there are nearly 200 *POR* mutations and single nucleotide polymorphisms (SNPs) [24], including more than 60 missense mutations (http://www.cypalleles.ki.se/por.htm). According to the cases reported in the literature, the most common mutation type in the Japanese population is R457H (51/78, 65.4%), and it is A287P (39/92, 42.4%) in Europeans [6, 11, 13, 16, 25–33], which shows significant ethnic differences. R457H was once thought to be a founder mutation in Japan [34], but this mutation has also been reported in Europeans and other ethnicity [35]. There are few PORD case reports in China. By searching the literature published in Chinese, we found that only two PORD cases have been reported, and both cases were compound heterozygous carriers including R457H. The patient we report here is the third Chinese PORD patient to be described, and she is homozygous for R457H, with parents who are heterozygous, nonconsanguineous carriers. These genetic characters suggest R457H maybe common in the Chinese PORD patients, which are similar with Japanese.

## Conclusions

In conclusion, PORD is a very rare disease which can be easily misdiagnosed in mild cases. Clinicians should keep in mind of this disease in patients with sexual dysfunction, especially combined with special skeletal deformities. Our data could provide a consciously understanding of this disease for clinic practicers. Low-dose corticosteroids combined with estrogen/progesterone sequential therapy will be effective in PORD patients with recurrence of large ovary cyst. The fact that the reported PORD patients in China carrying an identical variant R457H in *POR* gene also give us a viewpoint that R457H mutation in POR gene maybe also important in causing PORD in Chinese, which is similar with Japanese.

## Abbreviations

17-OHD: 17α hydroxylase deficiency
17OHP: 17-hydroxy-progesterone
21-OHD: 21-hydroxylase deficiency
ABS: Antley–Bixler syndrome
CAH: Congenital adrenal hyperplasia
CT: Computer tomography
PCOS: Polycystic ovary syndrome
POR: Cytochrome P450 oxidoreductase
PORD: Cytochrome P450 oxidoreductase deficiency
